# Factors associated with awareness of and willingness to use PrEP among stable, heterosexual HIV‐serodifferent couples in seven African countries, 2019–2022

**DOI:** 10.1002/jia2.26446

**Published:** 2025-06-18

**Authors:** J. Danielle Sharpe, Rebecca L. Laws, Christine A. West, Gaston Djomand, Jared Omolo, Dinah Ramaabya, Michelle Li, Sindisiwe Dlamini, Maletsatsi Motebang, Nthuseng Marake, Victor Singano, Washington Ozituosauka, Carter McCabe, Isabel Sathane, Nzali Kancheya, Tina Chisenga, Rickie Malaba, Getrude Ncube, Neena M. Philip, Samuel Biraro, Man E. Charurat, Italia Rolle, Andrew C. Voetsch

**Affiliations:** ^1^ Division of Global HIV and TB US Centers for Disease Control and Prevention Atlanta Georgia USA; ^2^ Epidemic Intelligence Service US Centers for Disease Control and Prevention Atlanta Georgia USA; ^3^ Division of Global HIV and TB US Centers for Disease Control and Prevention Gaborone Botswana; ^4^ Ministry of Health Gaborone Botswana; ^5^ Division of Global HIV and TB US Centers for Disease Control and Prevention Mbabane Eswatini; ^6^ Ministry of Health Mbabane Eswatini; ^7^ Division of Global HIV and TB US Centers for Disease Control and Prevention Maseru Lesotho; ^8^ Ministry of Health Maseru Lesotho; ^9^ Division of Global HIV and TB US Centers for Disease Control and Prevention Lilongwe Malawi; ^10^ Ministry of Health Lilongwe Malawi; ^11^ Division of Global HIV and TB US Centers for Disease Control and Prevention Maputo Mozambique; ^12^ Ministry of Health Maputo Mozambique; ^13^ Division of Global HIV and TB US Centers for Disease Control and Prevention Lusaka Zambia; ^14^ Ministry of Health Lusaka Zambia; ^15^ Division of Global HIV and TB US Centers for Disease Control and Prevention Harare Zimbabwe; ^16^ Ministry of Health and Child Care Harare Zimbabwe; ^17^ ICAP at Columbia University New York New York USA; ^18^ ICAP at Columbia University Kampala Uganda; ^19^ Center for International Health, Education, and Biosecurity University of Maryland School of Medicine Baltimore Maryland USA

**Keywords:** Africa, HIV, HIV‐serodifferent couples, Population‐based HIV Impact Assessment, pre‐exposure prophylaxis

## Abstract

**Introduction:**

HIV pre‐exposure prophylaxis (PrEP) is an effective biomedical intervention for preventing HIV; however, PrEP adoption initially lagged across sub‐Saharan Africa (SSA) and may have been affected by barriers to engagement in PrEP care. Stable, heterosexual HIV‐serodifferent couples are a priority population of PrEP expansion efforts. We assessed factors associated with PrEP awareness and willingness among HIV‐serodifferent couples in SSA to guide PrEP interventions for this population.

**Methods:**

We conducted a cross‐sectional analysis using pooled data from nationally representative, two‐stage cluster sampling, HIV‐focused household surveys completed during 2019–2022 in seven African countries. We analysed data from 1738 persons without HIV aged ≥15 years in stable, heterosexual HIV‐serodifferent couples and included clinical information from their partners with HIV. Higher HIV risk was defined by unawareness of a partner's HIV‐positive status or having a partner with an unsuppressed viral load (≥200 copies/ml). Lower HIV risk was defined by awareness of a partner's HIV‐positive status and having a partner with a suppressed viral load (<200 copies/ml). We conducted multivariable logistic regression using survey weights and jackknife variance estimation to assess factors associated with PrEP awareness and willingness.

**Results:**

Overall, 18.1% were aware of PrEP, 69.1% were willing to use PrEP and 5.1% had ever used PrEP. Forty‐four percent had higher HIV risk. Higher odds of PrEP awareness were associated with being female (adjusted odds ratio [aOR]: 1.73; 95% confidence interval [CI]: 1.15–2.59), secondary education or higher (aOR: 6.42; 95% CI: 2.97–13.91) and lower HIV risk (aOR: 1.58; 95% CI: 1.00–2.48). Higher odds of PrEP willingness were associated with employment in the past year (aOR: 1.55; 95% CI: 1.01–2.37), previous PrEP awareness (aOR: 2.44; 95% CI: 1.36–4.36) and lower HIV risk (aOR: 1.70; 95% CI: 1.07–2.70).

**Conclusions:**

Persons in stable, heterosexual HIV‐serodifferent couples with lower HIV risk were more aware of and willing to use PrEP than those with higher risk. Our findings highlight the importance of encouraging HIV status disclosure, educating about HIV‐serodifference and PrEP, and providing PrEP linkage during HIV testing and prevention counselling to increase PrEP awareness, willingness and use among HIV‐serodifferent couples in SSA.

## 1 INTRODUCTION

The global HIV epidemic disproportionately affects sub‐Saharan Africa (SSA), with an estimated 25.6 million people with HIV as of 2022, or two‐thirds of all people living with HIV globally [1]. SSA also accounted for approximately half of the 1.3 million new HIV acquisitions globally in 2022 [1]. Transmission among HIV‐serodifferent couples, which are defined as stable couples where one partner has HIV and the other does not, is an important source of incident HIV acquisitions in SSA [2, 3, 4], and an estimated 30% of total HIV incidence in SSA has been attributed to transmission among HIV‐serodifferent couples [5, 6]. Thus, HIV‐serodifferent couples are a priority population in SSA for HIV services, and there is a need to expand HIV prevention programmes with an emphasis on this population, including programmes improving the uptake of HIV pre‐exposure prophylaxis (PrEP).

Daily oral PrEP is a highly effective biomedical intervention for preventing HIV, reducing the risk of HIV transmission from sex by up to 99% [7, 8, 9, 10, 11]. In 2015, the World Health Organization (WHO) recommended that PrEP as part of combination HIV prevention services be offered to all persons at substantial risk of HIV, including HIV‐serodifferent couples when the partner with HIV does not have a suppressed viral load [12]. In 2016, the U.S. President's Emergency Plan for AIDS Relief (PEPFAR) began supporting PrEP implementation in SSA; however, the adoption of PrEP varied by country and initially lagged in the region [13, 14]. For instance, Zimbabwe [15], Lesotho [16] and Zambia [17] began introducing oral PrEP starting in 2016, Eswatini [18] and Botswana [19] in 2017, and Mozambique [20] and Malawi in 2018 [21]. Globally, persons in SSA represented an estimated 4% of all PrEP users in 2016, 31% of PrEP users in 2018, 59% of PrEP users in 2020 and 78% of PrEP users in 2022 [22].

Slower PrEP adoption and uptake in African countries may be affected by factors that serve as barriers to engagement in the PrEP care cascade, which includes PrEP awareness, willingness and use. Key factors include HIV status disclosure within HIV‐serodifferent couples and viral load suppression of the partner with HIV. HIV status disclosure and awareness of a partner's HIV status support engagement with risk reduction methods, such as PrEP, to prevent HIV acquisition for HIV‐serodifferent couples [23, 24, 25]. Antiretroviral therapy (ART) adherence and viral load suppression of the partner with HIV can affect engagement in PrEP care for the partner without HIV [25, 26, 27]. For couples where the partner with HIV is on ART and has a suppressed viral load, the risk of HIV transmission is negligible, as emphasized in the prevention communication strategy “Undetectable equals Untransmittable” or U = U [28]. Thus, decisions to use PrEP may be affected by partners’ initiation and adherence to ART, access to viral load monitoring, confidence in U = U and treatment as prevention, or perceived risk of HIV. Social determinants of health, defined by WHO as “conditions in which people are born, grow, work, live, and age,” [29, 30, 31, 32] such as educational attainment, stigma and discrimination, and access to PrEP, may also affect engagement in PrEP care.

Research on factors influencing engagement in PrEP care among HIV‐serodifferent couples in SSA is limited to certain countries and lacks population‐based data [33, 34]. We assessed factors associated with PrEP awareness and willingness among persons without HIV in stable, heterosexual HIV‐serodifferent couples using data from Population‐based HIV Impact Assessment (PHIA) surveys from seven African countries.

## 2 METHODS

### 2.1 Study design and analytical sample

We conducted an analysis of PrEP awareness, willingness and use using pooled data from seven PHIA surveys conducted in Botswana (2021), Eswatini (2021), Lesotho (2019–2020), Malawi (2020–2021), Mozambique (2021–2022), Zambia (2021) and Zimbabwe (2019–2020). Data from these seven surveys were included due to the availability of data on PrEP outcomes at the time of the analysis. PHIA surveys are cross‐sectional, nationally representative, household‐based, HIV‐focused surveys that use a two‐stage cluster sampling design to provide population‐level data on HIV indicators to assess the status of the HIV epidemic and the impact of national HIV response efforts in each country [35]. PHIA surveys include adults aged ≥15 years and consist of interviewer‐administered questionnaires that collect demographic, socio‐economic, behavioural and clinical information from participants. Home‐based HIV rapid testing and counselling are conducted according to each country's national testing algorithm, with laboratory confirmation and HIV viral load testing performed for HIV‐seropositive specimens [36].

Our study population included PHIA survey participants without HIV aged ≥15 years in stable, heterosexual HIV‐serodifferent couples. In total, there were 116,800 survey participants who were aged ≥15 years and had a laboratory blood test with a definitive HIV status determination. We restricted to 33,513 persons who self‐reported having any sexual partner who lived in the same household (stable partnerships) and then restricted to 32,741 persons who self‐reported having only one sexual partner who resided in the same household (couples). We further restricted to HIV‐serodifferent couples, specifically 1742 persons without HIV, as determined by household‐based HIV testing, who had a household sexual partner who was laboratory‐confirmed to have HIV. Then, we restricted our study population to HIV‐serodifferent couples who were determined to be heterosexual based on the self‐reported sex of each partner on the PHIA questionnaire for a total of 1738 persons without HIV who were in stable, heterosexual HIV‐serodifferent couples. Dyads of stable sexual partners not residing in the same household, polygamous relationships and same‐sex couples were excluded.

Using PHIA questionnaire data, we obtained information reported by participants without HIV in stable, heterosexual HIV‐serodifferent couples on demographic and socio‐economic characteristics and HIV‐related measures, such as sexual behaviours, prior HIV testing and diagnosis, self‐reported HIV status, and awareness, willingness and use of PrEP. We also obtained clinical information on viral load for partners with HIV in the identified couples.

### 2.2 Measures

PrEP outcomes included self‐reported awareness of PrEP, willingness to use PrEP and ever use of PrEP by persons without HIV in stable, heterosexual HIV‐serodifferent couples. During each PHIA survey, study staff described PrEP in the questionnaire to survey participants as: “‘PrEP’ or pre‐exposure prophylaxis, involves taking a daily pill to reduce the chance of getting HIV.” All persons who reported not having HIV were asked whether they had heard of PrEP prior to the survey (awareness). Persons who were aware of PrEP were asked whether they had ever taken PrEP (ever use). Persons who had not taken PrEP were asked if they would take PrEP to help prevent HIV, including those who only learned about PrEP during the interview. We defined PrEP willingness as those who had reported ever taking PrEP or those who reported they would take PrEP to help prevent HIV. We categorized all PrEP outcomes as yes or no.

Explanatory variables of interest were age, sex, educational attainment, employment status in the past 12 months, geography of residence (urban/rural) and country of residence. We included an asset‐based global wealth index, which was developed by pooling household wealth data from all countries included in the study [37]. We also assessed age difference within couples, condom use during the last sexual encounter, number of sexual partners in the past 12 months, awareness of the partner's HIV status and viral load suppression of the partner with HIV (HIV RNA <200 copies/ml). Awareness of HIV status was defined by whether the person without HIV accurately reported the HIV status of their partner with HIV. As a proxy for the U = U concept, we derived a measure of HIV risk by combining awareness of the partner's HIV status and viral load suppression. Higher HIV risk was defined as having had a partner with an unsuppressed viral load or having unawareness of their partner's HIV‐positive status. Lower HIV risk was defined as having had a partner with a suppressed viral load and awareness of their partner's HIV‐positive status.

### 2.3 Statistical analysis

The analysis was guided by multilevel conceptual frameworks highlighting individual and contextual factors associated with HIV disparities [38, 39, 40]. Using survey weights and jackknife variance estimation, we calculated weighted descriptive statistics, unadjusted odds ratios (ORs) and adjusted odds ratios (aORs) with 95% confidence intervals (CIs) using logistic regression models to assess factors associated with PrEP awareness and willingness. Age, sex, country of residence and survey year were a priori variables included in all adjusted models. Additional variables included in adjusted models were determined by bivariate analyses with a *p* value <0.20. The adjusted model for PrEP awareness additionally included educational attainment, employment status, wealth, geography of residence, condom use during the last sex and HIV risk status. The adjusted model for PrEP willingness additionally included age difference within couples, educational attainment, employment status, condom use during the last sex, number of sexual partners, HIV risk status and PrEP awareness. Due to the small sample size, we were unable to assess factors associated with PrEP use. All analyses were conducted using SAS 9.4 (SAS Institute, Cary, NC).

### 2.4 Ethics statement

The PHIA surveys were funded by PEPFAR with technical assistance through the U.S. Centers for Disease Control and Prevention (CDC) under the terms of the cooperative agreements #U2GGH002173 and #U2GGH002172. Each PHIA survey was reviewed and approved by human subject institutional review boards at CDC, local ethics boards in each country and ICAP at Columbia University or the University of Maryland, Baltimore.^1^ All participants provided informed consent prior to participating in a PHIA survey. Minors aged 15–17 years provided assent after parental permission was obtained.

## 3 RESULTS

There were 1738 persons without HIV who were in stable, heterosexual HIV‐serodifferent couples (Table 1). The median age was 36 years (interquartile range: 28–45 years). Of these persons, more than half were men (55.0%), 50.9% had a primary education, 51.7% were unemployed in the past 12 months and 56.1% were in the upper 60% of household wealth. Approximately two‐thirds resided in a rural area (65.4%), 63.5% were aware of their partner's HIV‐positive status and 69.9% had a partner with a suppressed viral load. Also, 77.2% did not use a condom during the last sex, 87.4% reported having only one sexual partner in the past 12 months and 44.0% demonstrated higher HIV risk.

**Table 1:** Characteristics of persons without HIV in HIV‐serodifferent couples—seven Population‐based HIV Impact Assessments, 2019–2022

	Overall (*N* = 1738)		PrEP awareness				PrEP willingness			
	Weighted %	Total	Yes Weighted %	Total	No Weighted %	Total	Yes Weighted %	Total	No Weighted %	Total
Characteristics	(95% CI)		(95% CI)		(95% CI)		(95% CI)		(95% CI)	
**Total**		1738	18.1 (15.3−20.9)	476	81.9 (79.1−84.7)	1262	69.1 (65.1−73.2)	1252	30.9 (26.8−34.9)	486
**Age (years)**										
15–34	42.2 (38.2−46.3)	590	16.6 (12.6−20.5)	156	83.4 (79.5−87.4)	434	68.1 (62.1−74.0)	438	31.9 (26.0−37.9)	152
35–44	30.2 (26.6−33.7)	547	20.6 (15.4−25.9)	163	79.4 (74.1−84.6)	384	69.8 (63.1−76.5)	399	30.2 (23.5−36.9)	148
45+	27.6 (24.8−30.3)	601	17.7 (13.8−21.5)	157	82.3 (78.5−86.2)	444	70.0 (64.2−75.9)	415	30.0 (24.2−35.8)	186
**Age difference within couples (years)**										
<5	39.5 (35.3−43.6)	665	16.9 (12.9−20.9)	174	83.1 (79.1−87.1)	491	70.6 (64.8−76.3)	484	29.4 (23.7−35.2)	181
5–9	31.6 (27.6−35.5)	568	21.0 (15.8−26.1)	170	79.0 (73.9−84.2)	398	70.9 (64.2−77.6)	418	29.1 (22.4−35.8)	150
10+	29.0 (25.9−32.1)	505	16.6 (11.7−21.5)	132	83.4 (78.5−88.3)	373	65.2 (58.9−71.6)	350	34.8 (28.4−41.1)	155
**Sex**										
Male	55.0 (51.4−58.6)	931	17.6 (13.8−21.4)	229	82.4 (78.6−86.2)	702	72.9 (67.2−78.6)	684	27.1 (21.4−32.8)	247
Female	45.0 (41.4−48.6)	807	18.7 (14.7−22.7)	247	81.3 (77.3−85.3)	560	64.5 (58.2−70.9)	568	35.5 (29.1−41.8)	239
**Educational attainment**										
No education	14.1 (10.7−17.4)	203	4.2 (1.6−6.8)	24	95.8 (93.2−98.4)	179	55.3 (44.5−66.1)	132	44.7 (33.9−55.5)	71
Primary	50.9 (47.1−54.8)	802	13.9 (10.2−17.6)	179	86.1 (82.4−89.8)	623	69.6 (63.6−75.7)	582	30.4 (24.3−36.4)	220
Secondary or higher	35.0 (31.3−38.7)	733	29.8 (24.8−34.8)	273	70.2 (65.2−75.2)	460	73.9 (69.3−78.6)	538	26.1 (21.4−30.7)	195
**Employment status in the past 12 months**										
Employed	48.3 (44.6−52.1)	793	20.2 (15.8−24.6)	236	79.8 (75.4−84.2)	557	76.2 (71.0−81.5)	605	23.8 (18.5−29.0)	188
Unemployed	51.7 (47.9−55.4)	945	16.2 (12.6−19.7)	240	83.8 (80.3−87.4)	705	62.5 (57.3−67.7)	647	37.5 (32.3−42.7)	298
**Household wealth index**										
Lower 40%	43.9 (39.9−47.9)	622	12.9 (9.2−16.6)	101	87.1 (83.4−90.8)	521	67.9 (61.8−73.9)	460	32.1 (26.1−38.2)	162
Upper 60%	56.1 (52.1−60.1)	1116	22.2 (18.4−26.0)	375	77.8 (74.0−81.6)	741	70.1 (65.2−75.0)	792	29.9 (25.0−34.8)	324
**Geography of residence**										
Urban	34.6 (29.5−39.6)	575	22.4 (17.7−27.0)	184	77.6 (73.0−82.3)	391	66.8 (59.4−74.1)	383	33.2 (25.9−40.6)	192
Rural	65.4 (60.4−70.5)	1163	15.9 (12.5−19.2)	292	84.1 (80.8−87.5)	871	70.4 (65.1−75.7)	869	29.6 (24.3−34.9)	294
**Awareness of partner's HIV‐positive status**										
Aware	63.5 (59.8−67.3)	983	25.7 (21.2−30.3)	343	74.3 (69.7−78.8)	640	76.4 (71.7−81.0)	746	23.6 (19.0−28.3)	237
Unaware	36.5 (32.7−40.2)	389	17.1 (10.7−23.4)	79	82.9 (76.6−89.3)	310	65.8 (57.8−73.8)	259	34.2 (26.2−42.2)	130
**Condom use during last sexual encounter**										
Yes	22.8 (20.4−25.3)	607	25.2 (20.0−30.4)	210	74.8 (69.6−80.0)	397	73.6 (67.3−80.0)	440	26.4 (20.0−32.7)	167
No	77.2 (74.7−79.6)	1127	16.0 (12.9−19.2)	266	84.0 (80.8−87.1)	861	67.7 (63.0−72.5)	811	32.3 (27.5−37.0)	316
**Number of sexual partners in the past 12 months**										
1	87.4 (84.7−90.0)	1554	18.0 (15.0−20.9)	419	82.0 (79.1−85.0)	1135	67.7 (63.3−72.2)	1107	32.3 (27.8−36.7)	447
2+	12.6 (10.0−15.3)	184	19.0 (10.4−27.6)	57	81.0 (72.4−89.6)	127	78.8 (68.3−89.3)	145	21.2 (10.7−31.7)	39
**Viral load suppression status (<200 copies/ml) of partner with HIV**										
Suppressed	69.9 (66.1−73.7)	1354	21.2 (17.6−24.8)	386	78.8 (75.2−82.4)	968	71.1 (67.4−74.9)	986	28.9 (25.1−32.6)	368
Unsuppressed	30.1 (26.3−33.9)	377	11.0 (7.3−14.8)	87	89.0 (85.2−92.7)	290	64.9 (56.2−73.5)	262	35.1 (26.5−43.8)	115
**HIV risk status** ^a^										
Higher HIV risk	44.0 (39.9−48.0)	465	17.7 (12.2−23.2)	107	82.3 (76.8−87.8)	358	68.0 (59.1−76.8)	317	32.0 (23.2−40.9)	148
Lower HIV risk	56.0 (52.0−60.1)	900	26.5 (21.6−31.5)	312	73.5 (68.5−78.4)	588	76.4 (71.9−81.0)	684	23.6 (19.0−28.1)	216
**PrEP awareness prior to the survey**										
Aware of PrEP	18.1 (15.3−20.9)	476	−	−	−	−	83.2 (77.1−89.3)	376	16.8 (10.7−22.9)	100
Unaware of PrEP	81.9 (79.1−84.7)	1262	−	−	−	−	66.0 (61.5−70.6)	876	34.0 (29.4−38.5)	386

*Note*: Population‐based HIV Impact Assessments were conducted in Botswana (2021), Eswatini (2021), Lesotho (2019–2020), Malawi (2020–2021), Mozambique (2021–2022), Zambia (2021) and Zimbabwe (2019–2020).

Abbreviations: CI, confidence interval; PrEP, pre‐exposure prophylaxis.

^a^ Higher HIV risk was defined by whether persons without HIV were unaware of their partner's HIV‐positive status or had a partner with an unsuppressed viral load. Lower HIV risk was defined by whether persons without HIV both had a partner with a suppressed viral load and were aware of their partner's HIV‐positive status.

**Figure 1:**
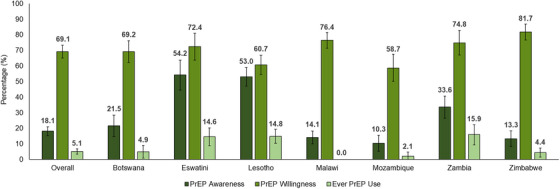
Awareness, willingness and use of HIV pre‐exposure prophylaxis (PrEP) among persons without HIV in HIV‐serodifferent couples overall and by country—seven Population‐based HIV Impact Assessments, 2019–2022.

Overall, 18.1% (95% CI: 15.3–20.9) were aware of PrEP, 69.1% (95% CI: 65.1–73.2) were willing to use PrEP and 5.1% (95% CI: 3.4–6.8) had ever used PrEP (Figure 1). There were differences in PrEP awareness, willingness and use by country. PrEP awareness ranged from 10.3% (95% CI: 5.2–15.4) in Mozambique to 54.2% (95% CI: 44.6–63.8) in Eswatini. PrEP willingness ranged from 58.7% (95% CI: 50.1–67.4) in Mozambique to 81.7% (95% CI: 76.6–86.9) in Zimbabwe. Ever PrEP use ranged from 0% (95% CI: 0–0) in Malawi to 15.9% (95% CI: 9.4–22.3) in Zambia. Also, 83.2% (95% CI: 77.1–89.3) of persons who were aware of PrEP prior to the PHIA survey were willing to use PrEP, and 66.0% (95% CI: 61.5–70.6) of persons who were unaware of PrEP prior to the survey were willing to use PrEP.

In adjusted analyses, higher odds of PrEP awareness were associated with being female compared to being male (aOR: 1.73; 95% CI: 1.15–2.59) (Table 2). Persons who reported having a primary education (aOR: 2.55; 95% CI: 1.12–5.81) or secondary education or higher (aOR: 6.42; 95% CI: 2.97–13.91) had higher odds of PrEP awareness than persons with no education. Persons with lower HIV risk had higher odds of PrEP awareness than those with higher HIV risk (aOR: 1.58; 95% CI: 1.00–2.48).

**Table 2:** Factors associated with PrEP awareness among persons without HIV in HIV‐serodifferent couples—seven Population‐based HIV Impact Assessments, 2019–2022

	Unadjusted model	Adjusted model
Characteristics	OR (95% CI)	aOR % (95% CI)^a^
**Age (years)**		
15–34	Ref	Ref
35–44	1.31 (0.89−1.93)^*^	1.37 (0.84−2.25)
45+	1.08 (0.75−1.55)	1.03 (0.64−1.65)
**Age difference within couples (years)**		
<5	1.02 (0.66−1.59)	−
5–9	1.33 (0.82−2.16)	−
10+	Ref	−
**Sex**		
Male	Ref	Ref
Female	1.08 (0.75−1.55)	1.73 (1.15−2.59)^**^
**Educational attainment**		
No education	Ref	Ref
Primary	3.64 (1.77−7.49)^*^	2.55 (1.12−5.81)^**^
Secondary or higher	9.58 (4.73−19.39)^*^	6.42 (2.97−13.91)^**^
**Employment status in the past 12 months**		
Employed	1.31 (0.90−1.92)^*^	1.29 (0.82−2.03)
Unemployed	Ref	Ref
**Household Wealth Index**		
Lower 40%	Ref	Ref
Upper 60%	1.92 (1.32−2.81)^*^	1.05 (0.67−1.63)
**Geography of residence**		
Urban	1.53 (1.06−2.20)^*^	1.15 (0.72−1.84)
Rural	Ref	Ref
**Condom use during last sexual encounter**		
Yes	1.77 (1.26−2.48)^*^	1.16 (0.79−1.70)
No	Ref	Ref
**Number of sexual partners in the past 12 months**		
1	Ref	−
2+	1.07 (0.60−1.91)	−
**HIV risk status** ^b^		
Higher HIV risk	Ref	Ref
Lower HIV risk	1.68 (1.07−2.62)^*^	1.58 (1.00−2.48)^**^

*Note*: Population‐based HIV Impact Assessments were conducted in Botswana (2021), Eswatini (2021), Lesotho (2019–2020), Malawi (2020–2021), Mozambique (2021–2022), Zambia (2021) and Zimbabwe (2019–2020).

Abbreviations: aOR, adjusted odds ratio; CI, confidence interval; OR, odds ratio; PrEP, pre‐exposure prophylaxis.

^a^ Adjusted for age, sex, educational attainment, employment status in the past 12 months, wealth, geography of residence, condom use during last sexual encounter, HIV risk status, country of residence and survey year.

^b^ Higher HIV risk was defined by whether persons without HIV were unaware of their partner's HIV‐positive status or had a partner with an unsuppressed viral load. Lower HIV risk was defined by whether persons without HIV both had a partner with a suppressed viral load and were aware of their partner's HIV‐positive status.

* *p* <0.20.

** *p* <0.05.

In adjusted analyses, higher odds of PrEP willingness were associated with being employed in the past 12 months than being unemployed in the past 12 months (aOR: 1.55; 95% CI: 1.01–2.37) (Table 3). Having a less than 5‐year age difference within HIV‐serodifferent couples was associated with higher odds of PrEP willingness compared to having a 10+ year age difference within couples (aOR: 1.85; 95% CI: 1.11–3.06). Persons with lower HIV risk had higher odds of PrEP willingness than those with higher HIV risk (aOR: 1.70; 95% CI: 1.07–2.70). Those who were previously aware of PrEP had higher odds of PrEP willingness than those who learned about PrEP during the survey (aOR: 2.44; 95% CI: 1.36–4.36).

**Table 3:** Factors associated with PrEP willingness among persons without HIV in HIV‐serodifferent couples—seven Population‐based HIV Impact Assessments, 2019–2022

	Unadjusted model	Adjusted model
Characteristics	OR (95% CI)	aOR % (95% CI)^a^
**Age (years)**		
15–34	Ref	Ref
35–44	1.09 (0.78−1.52)	0.69 (0.43−1.11)
45+	1.10 (0.74−1.63)	0.86 (0.51−1.44)
**Age difference within couples (years)**		
<5	1.28 (0.89−1.83)^*^	1.85 (1.11−3.06)^**^
5–9	1.30 (0.88−1.92)^*^	1.09 (0.68−1.77)
10+	Ref	Ref
**Sex**		
Male	1.48 (0.97−2.26)^*^	1.24 (0.78−1.97)
Female	Ref	Ref
**Educational attainment**		
No education	Ref	Ref
Primary	1.85 (1.09−3.13)^*^	1.18 (0.66−2.08)
Secondary or higher	2.29 (1.38−3.82)^*^	1.06 (0.56−1.99)
**Employment status in the past 12 months**		
Employed	1.93 (1.37−2.71)^*^	1.55 (1.01−2.37)^**^
Unemployed	Ref	Ref
**Household Wealth Index**		
Lower 40%	Ref	−
Upper 60%	1.11 (0.79−1.56)	−
**Geography of residence**		
Urban	Ref	−
Rural	1.18 (0.76−1.84)	−
**Condom use during last sexual encounter**		
Yes	1.33 (0.91−1.94)^*^	0.98 (0.60−1.59)
No	Ref	Ref
**Number of sexual partners in the past 12 months**		
1	Ref	Ref
2+	1.77 (0.88−3.58)^*^	1.47 (0.62−3.50)
**HIV risk status** ^b^		
Higher HIV risk	Ref	Ref
Lower HIV risk	1.53 (0.95−2.45)^*^	1.70 (1.07−2.70)^*^
**PrEP awareness prior to the survey**		
Aware of PrEP	2.55 (1.58−4.10)^*^	2.44 (1.36−4.36)^**^
Unaware of PrEP	Ref	Ref

*Note*: Population‐based HIV Impact Assessments were conducted in Botswana (2021), Eswatini (2021), Lesotho (2019–2020), Malawi (2020–2021), Mozambique (2021–2022), Zambia (2021) and Zimbabwe (2019–2020).

Abbreviations: aOR, adjusted odds ratio; CI, confidence interval; OR, odds ratio; PrEP, pre‐exposure prophylaxis.

^a^ Adjusted for age, age difference within couples, sex, educational attainment, employment status in the past 12 months, condom use during last sexual encounter, number of sexual partners in the past 12 months, HIV risk status, PrEP awareness, country of residence and survey year.

^b^ Higher HIV risk was defined by whether persons without HIV were unaware of their partner's HIV‐positive status or had a partner with an unsuppressed viral load. Lower HIV risk was defined by whether persons without HIV both had a partner with a suppressed viral load and were aware of their partner's HIV‐positive status.

* *p* <0.20.

** *p* <0.05.

## 4 DISCUSSION

Although PrEP use was low and awareness of PrEP varied across these countries, we found that more than two‐thirds of persons without HIV in HIV‐serodifferent couples reported willingness to use PrEP. This finding signifies that additional efforts to improve PrEP literacy and facilitate access to PrEP may be warranted, such as integrating PrEP education and delivery into routine individual and couples‐focused sexual health services [41, 42, 43, 44]. Additionally, we found country‐specific differences in PrEP awareness, willingness and use. Eswatini and Zambia had the highest prevalence of PrEP awareness, willingness and use. These countries are characterized by mature generalized HIV epidemics, being on track to achieve or having achieved epidemic control based on the UNAIDS 95–95–95 targets, and early introductions of PrEP among priority populations such as HIV‐serodifferent couples, which contributed to greater availability of PrEP [14, 45, 46, 47, 48]. Nonetheless, overall PrEP use across all seven African countries has yet to reach coverage levels needed to achieve the UNAIDS PrEP target of at least 30% of persons without HIV in HIV‐serodifferent partnerships using PrEP [49].

We found that persons without HIV in HIV‐serodifferent couples who had lower HIV risk reported higher levels of PrEP awareness and willingness than those with higher HIV risk. This indicates there may be a need to improve PrEP awareness and willingness among couples with higher HIV risk. This finding illustrates the importance of increasing PrEP awareness and availability, normalizing PrEP as part of routine sexual health services and encouraging informed choice for all persons regardless of HIV risk. Persons with higher HIV risk may not experience the social support needed to engage in PrEP care [50, 51], further highlighting the importance of different approaches such as empowering persons with HIV to practice safer sex and voluntarily disclose their HIV status in safe environments. For example, research from Uganda demonstrated how HIV status disclosure helped establish perceived HIV risk to facilitate the acceptability of PrEP as a method for preventing HIV among HIV‐serodifferent couples [23, 24]. Also, studies from Kenya and Uganda demonstrated that some persons in HIV‐serodifferent couples who have lower HIV risk continue using PrEP even once their partner has a suppressed viral load because of the anticipated increased risk of HIV if PrEP is discontinued or a lack of trust in U = U [27, 52].

PrEP awareness was strongly associated with PrEP willingness in our study. This relationship is plausible considering the importance of awareness and knowledge of PrEP in the development of motivation and willingness to take PrEP [53]. This finding is consistent with studies conducted in South Africa [54], Eswatini [55] and Uganda [56] reporting that higher levels of PrEP awareness contributed to higher levels of willingness or intention to use PrEP. Interestingly, although we found that PrEP willingness was higher among persons in HIV‐serodifferent couples who were previously aware of PrEP, two‐thirds of persons in HIV‐serodifferent couples who learned of PrEP during the survey demonstrated willingness to use PrEP to prevent HIV. This highlights the opportunity to emphasize PrEP education efforts during individual and couples‐focused HIV testing and prevention counselling to increase PrEP awareness and improve PrEP willingness among persons without HIV in HIV‐serodifferent couples disproportionately affected by HIV. This may also include routine provider follow‐up with persons with a detectable viral load to increase awareness of the benefits of PrEP to prevent HIV transmission to their partner without HIV, including promoting U = U as part of a comprehensive prevention package. Counselling sessions with trained staff can provide support to HIV‐serodifferent couples to encourage engagement in HIV risk reduction during periods of heightened transmission, in addition to counselling HIV‐serodifferent couples who may opt to use PrEP or other prevention methods for different reasons [57, 58].

Additionally, social determinants of health were associated with PrEP awareness and willingness among HIV‐serodifferent couples in our study. Being female was associated with greater PrEP awareness, signifying that barriers to PrEP awareness for men may need to be addressed [59]. This finding is consistent with research conducted in South Africa highlighting that young women reported higher levels of PrEP awareness than young men [54] as well as research from Tanzania [60] and Kenya [61] demonstrating high awareness of PrEP among women who perceived themselves to be at increased risk of acquiring HIV. Because younger women consistently have high HIV incidence in SSA, they are generally a primary focus of PrEP‐related interventions in this region, which may explain their higher PrEP awareness. Also, higher levels of educational attainment were associated with greater PrEP awareness, which is consistent with studies from Malawi [62] and Cameroon [63]. This suggests that PrEP literacy efforts may need to be more accessible across educational levels. Moreover, we found that persons who were employed in the past year were more willing to use PrEP than those who were unemployed, suggesting PrEP initiatives might need to address inequities of persons with socio‐economic challenges who may experience barriers to accessing PrEP services. In addition, a less than 5‐year age difference within HIV‐serodifferent couples was associated with greater PrEP willingness compared to an age difference of 10+ years, which may be explained by the increased risk of HIV associated with age‐disparate sexual relationships and related power dynamics [64, 65, 66, 67, 68]. The disparities in PrEP awareness and willingness identified in our study may also be considered in light of additional inequities that disproportionately affect HIV‐serodifferent couples in SSA, such as economic disadvantage, sexual and intimate partner violence, and stigma and discrimination [1, 69].

To our knowledge, this is the first study to estimate the distribution and determinants of PrEP awareness and willingness among stable, heterosexual HIV‐serodifferent couples in multiple African countries. However, our study is subject to limitations. First, we defined HIV‐serodifferent couples as heterosexual dyads of sexual partners where a person without HIV and a person with laboratory‐confirmed HIV were stable sexual partners and resided in the same household. Our definition excluded dyads of stable sexual partners who do not reside within the same household, polygamous relationships and same‐sex couples. Along these lines, we were unable to include any measures of relationship duration or confirm the self‐reported number of sexual partners among PHIA participants. Second, our definition of higher HIV risk included those unaware of their partner's HIV status. This may be a limitation because their partner may have a suppressed viral load which may not represent HIV risk at the time of the PHIA survey; however, their partner may not maintain a suppressed viral load, and a lack of awareness of the potential for HIV transmission signifies risk for HIV acquisition. Third, we did not assess all factors that may affect engagement in PrEP care, such as stigma, PrEP accessibility, transportation access, and other social, economic and political factors. Fourth, the seven countries in the analysis are located in southern Africa, and therefore, the results may not be generalizable to all sub‐Saharan African countries. Lastly, due to the small sample size, we were unable to assess factors associated with PrEP use. We analysed data from seven PHIA surveys that were collected between 2019 and 2022, which may have occurred during or before national PrEP programmes were established in some countries. Also, these PHIA surveys overlapped with the COVID‐19 pandemic, which temporarily disrupted data collection for some surveys in 2020 and 2021. Despite these limitations, we presented robust baseline data on PrEP awareness, willingness, and use and examined factors associated with PrEP awareness and willingness among persons without HIV in HIV‐serodifferent couples from seven African countries that are disproportionately affected by the HIV epidemic.

Our findings have potential implications for guiding PrEP programmes tailored towards HIV‐serodifferent couples in SSA. Considering our findings on social determinants of health, PrEP programmes may tailor programming to better reach populations that encounter social challenges and inequities that may be barriers to PrEP care, such as socio‐economic disadvantage. For instance, in 42 countries with generalized HIV epidemics, including 36 African countries, cash transfer programmes led to improvements in HIV‐related outcomes possibly through offsetting costs associated with accessing HIV care services, which may extend to PrEP services as well [70]. Moreover, our findings suggest the importance of educating couples about HIV‐serodifference, U = U and PrEP, encouraging HIV status disclosure among couples, and jointly providing PrEP to the partner without HIV and ART to the partner with HIV with an unsuppressed viral load. Integrated HIV counselling sessions incorporating discussions about U = U, HIV status disclosure and PrEP‐ART initiation have been shown to maximize the delivery of PrEP among HIV‐serodifferent couples [41, 42, 43, 44, 57, 71, 72]. Studies from Kenya [73], Tanzania [51], South Africa [74], Uganda [75] and Eswatini [55] have also shown the importance of clinic‐based PrEP education programmes in improving PrEP awareness among the general population. PrEP programmes may benefit from expanding individual and couples‐focused HIV testing, PrEP education, and prevention counselling integrated with care and treatment, especially for educating HIV‐serodifferent couples about U = U and the benefits of PrEP and ART for preventing HIV and dually providing PrEP and ART to couples as needed.

## 5 CONCLUSIONS

Addressing barriers to the scale‐up of HIV services, including PrEP, is a priority of the UNAIDS strategy to end HIV as a global health threat by 2030. We conducted a cross‐sectional analysis to assess factors associated with engagement in the PrEP care cascade among 1738 persons without HIV in stable, heterosexual HIV‐serodifferent couples in Botswana, Eswatini, Lesotho, Malawi, Mozambique, Zambia and Zimbabwe. Our study suggests that although PrEP willingness is high, there is an opportunity to improve PrEP awareness and use among persons without HIV in HIV‐serodifferent couples. Findings from our study also highlight the importance of encouraging HIV status disclosure, educating individuals and couples at increased risk for HIV about HIV‐serodifference, U = U and PrEP, and integrating the delivery of PrEP and ART into prevention counselling and testing for HIV‐serodifferent couples. Expanding access to programmes that integrate PrEP‐ART education, provision and delivery for HIV‐serodifferent couples may support continued progress on reductions in HIV incidence and achievement of the UNAIDS 2030 targets in SSA.

## COMPETING INTERESTS

The authors declare that they have no competing interests.

## AUTHORS’ CONTRIBUTIONS

JDS, RLL, CAW and ACV conceptualized the study. JDS conducted the analysis and drafted the manuscript, with methodological and interpretation support from RLL, CAW and ACV. All authors reviewed, revised and approved the manuscript.

## FUNDING

This publication has been supported by the President's Emergency Plan for AIDS Relief (PEPFAR) through the Centers for Disease Control and Prevention (CDC) under the terms of the cooperative agreements #U2GGH002173 and #U2GGH002172.

## CDC DISCLAIMER

The findings and conclusions in this publication are those of the author(s) and do not necessarily represent the official position of the funding agencies.

## Data Availability

The data we report in this study are publicly available and/or available upon request from the Population‐based HIV Impact Assessment (PHIA) Project website: https://phia.icap.columbia.edu/.

## Footnotes

1 See 45 C.F.R. part 46; 21 C.F.R. part 56.
